# High efficiency electrotransformation of *Lactococcus lactis *spp. *lactis *cells pretreated with lithium acetate and dithiothreitol

**DOI:** 10.1186/1472-6750-7-15

**Published:** 2007-03-21

**Authors:** Maria Papagianni, Nicholaos Avramidis, George Filioussis

**Affiliations:** 1 Department of Hygiene and Technology, School of Veterinary Medicine, Aristotle University of Thessaloniki, Thessaloniki 54006, Greece

## Abstract

**Background:**

A goal for the food industry has always been to improve strains of *Lactococcus lactis *and stabilize beneficial traits. Genetic engineering is used extensively for manipulating this lactic acid bacterium, while electropolation is the most widely used technique for introducing foreign DNA into cells. The efficiency of electrotransformation depends on the level of electropermealization and pretreatment with chemicals which alter cell wall permeability, resulting in improved transformation efficiencies is rather common practice in bacteria as in yeasts and fungi. In the present study, treatment with lithium acetate (LiAc) and dithiothreitol (DTT) in various combinations was applied to *L. lactis *spp. *lactis *cells of the early-log phase prior to electroporation with plasmid pTRKH3 (a 7.8 kb shuttle vector, suitable for cloning into *L. lactis*). Two strains of *L. lactis *spp. *lactis *were used, *L. lactis *spp. *lactis *LM0230 and ATCC 11454. To the best of our knowledge these agents have never been used before with *L. lactis *or other bacteria.

**Results:**

Electrotransformation efficiencies of up to 10^5 ^transformants per μg DNA have been reported in the literature for *L. lactis *spp.*lactis *LM0230. We report here that treatment with LiAc and DDT before electroporation increased transformation efficiency to 225 ± 52.5 × 10^7 ^transformants per μg DNA, while with untreated cells or treated with LiAc alone transformation efficiency approximated 1.2 ± 0.5 × 10^5 ^transformants per μg DNA. Results of the same trend were obtained with *L. lactis *ATCC 11454, although transformation efficiency of this strain was significantly lower. No difference was found in the survival rate of pretreated cells after electroporation. Transformation efficiency was found to vary directly with cell density and that of 10^10 ^cells/ml resulted in the highest efficiencies. Following electrotransformation of pretreated cells with LiAc and DDT, pTRKH3 stability was examined. Both host-vector systems proved to be reproducible and highly efficient.

**Conclusion:**

This investigation sought to improve still further transformation efficiencies and to provide a reliable high efficiency transformation system for *L. lactis *spp. *lactis*. The applied methodology, tested in two well-known strains, allows the production of large numbers of transformants and the construction of large recombinant libraries.

## Background

*Lactococcus lactis *is the model lactic acid bacterium extensively used in the manufacture of fermented foods of animal origin. A goal for the food industry has always been to improve *L. lactis *strains and to stabilize important traits of this bacterium. Today, genetic engineering is used extensively for manipulating *L. lactis*. Important contributions to the evolution of genetic technology of this organism include the development of transformation techniques and the construction of powerful plasmids for gene cloning and for general mutagenesis [1,2]. In the past years, electroporation has become the widest used method for introducing DNA in *L. lactis *cells. Transformation by electroporation involves the application of a brief, high voltage electrical pulse to a suspension of cells and DNA. Although the molecular mechanism of electrotransformation is not completely understood, the electrical pulse is though to result in a rearrangement of the components of the cell wall and membrane to generate transient pores through which the DNA can pass into the cell [3].

Harlander [4] was the first to employ electroporation in *L. lactis*. Later, McIntyre and Harlander [5,6] improved electroporation efficiency of intact *L. lactis *cells by adjusting a number of parameters like the growth phase and final concentration of cells, the growth medium, the concentration of plasmid DNA and the amplitude and duration of the pulse. Since then, numerous papers describing the transformation of lactic acid bacteria have been published [3]. The efficiency of electrotransformation is strongly correlated to the level of electropermeabilization [7]. As the physical barrier of the cell wall has to be weakened enough in order that an adequate amount of DNA will enter the cell, pretreatment with chemicals that increase cell wall permeability, and subsequently improve the transformation rate, has often been proposed. Pretreatment with lysozyme has been proposed by Powel et al. [8], threonine, by Van der Lelie et al. [9] and Dornan and Collins [10] and glycine, by Holo and Nes [11] and Le Bourgeois et al. [12]. The effect of cell wall weakening agents, however, is not universal as they were found to be either highly strain-specific [13] or completely ineffective with some microorganisms [14].

Chemical treatment prior to electroporation tends to be common practice today with bacteria and fungi and various chemicals are proposed in electrotransformation protocols, while their mechanism of action is not always known. CaCl_2 _for example, is used successfully with *Escherichia coli *[15] and what makes the treatment successful is still unknown. Similarly, thiol compounds and lithium acetate are used successfully with yeasts while the way they act and increase transformation efficiencies remains unknown [16].

In the present work, the transformation efficiency of *L. lactis *spp.*lactis *LM0230 and *L. lactis *spp.*lactis *ATCC 11454 cells, treated with various combinations of LiAc and DTT before electroporation was examined. According to literature information, pretreatment with these chemicals has never been applied before with *L. lactis *or other bacteria. A tremendous improvement of transformation efficiency was observed in cells of both tested strains treated with both LiAc and DDT. The effect of cell density on transformation efficiency of pretreated cells was also studied.

## Results and discussion

McIntyre and Harlander [5,6] studied the influence of the growth phase of cells and cell density on transformation efficiency of *L. lactis *LM0230. Transformation efficiencies were significantly higher (up to 1 × 10^3 ^transformants/μg of DNA for 9.8 kb plasmid pGB301) using late stationary phase cells (OD_600 _= 1.2) at high cell concentrations (5 × 10^10 ^CFU/ml) when subjected to high voltage electric pulses. However, improved transformation efficiencies of approximately 10^5 ^transformants/μg DNA with the same system of host/vector were achieved by Dornan and Collins [10] by altering the conditions under which the bacteria were grown prior to electroporation, e.g. by incorporating threonine in the medium, while cells of the early-log phase were used. Le Bourgeois et al. [12] reported transformation efficiencies of 1–2 × 10^7 ^transformants/μg of DNA for *L. lactis *spp.*cremoris *MG1363 using pIL253 as plasmid DNA, and a medium containing glycine (20% w/v) and 0.5 M sucrose, as osmotic stabilizer.

In the present study, the conditions applied by Dornan and Collins [10] were mostly adopted and transformation efficiencies were determined for cells without and following pretreatment with various combinations of LiAc and DTT with *L. lactis *LM0230 and *L. lactis *ATCC 11454 and plasmid pTRKH3, a 7.8 kb plasmid which is a shuttle vector with a wide host range of Gram-positive bacterial strains including *L. lactis *[17]. LiAc is used widely in yeast transformation [18], while pretreatment with thiol compounds has also been applied [19]. Thomson et al. [20] pretreated *Saccharomyces cerevisiae *with LiAc and DTT and reported enhanced transformation efficiency. Wu and Lechworth [16] also used a combination of LiAc and DTT and achieved high efficiency transformation of *Pichia pastoris*, otherwise known for its lower transformation efficiencies compared to other yeasts. While the mechanism for these effects is unclear, treatment of *S. cerevisiae *with a combination of LiAc and a reducing agent has been shown to modify cell wall porosity and increase their permeability. To the best of our knowledge, the successfully applied to various yeasts treatments with LiAc and DTT, have never been tested before with bacteria.

Transformation efficiencies achieved with various treatments for the electroporation of plasmid pTRKH3 into *L. lactis *LM0230 and *L. lactis *ATCC 11454 are shown in Table 1. Plasmid analysis of 20 putative transformants from each strain, selected randomly from each trial revealed the presence of the plasmid pTRKH3 (which was absent from the *L. lactis *LM0230 strain). Untransformed cells failed to grow in antibiotic containing medium. The results presented in Table 1 show a tremendous increase in transformation efficiency in cells pretreated with both LiAc and DTT: In the case of *L. lactis *LM0230 for example, 225 ± 52.5 × 10^7 ^transformants/μg DNA (*P *< 0.001) with pretreated cells versus 1.20 ± 0.5 × 10^5 ^transformants/μg DNA in the case of cells directly electroporated or 1.10 ± 0.52 × 10^5 ^transformants/μg when cells were treated with LiAc alone. Treatment with DTT alone resulted in a significant improvement of transformation efficiency (13 ± 8.02 × 10^6 ^transformants/μg DNA). Results of the same trend were obtained with *L. lactis *ATCC 11454, although, as shown in Table 1, this strain shows significantly lower electrotransformation efficiency compared to *L. lactis *LM0230. Unlike with *L. lactis *LM0230, there are no reports in the literature to provide information on transformation efficiency of *L. lactis *ATCC 11454. It is well known however, that transformation efficiencies could vary widely between strains of the same species [12]. Obviously, the combination of both chemicals, which may act through different mechanisms, seem to multiply the effects. No difference was found in the survival rate of pretreated cells of both strains after electroporation. Under the particular tested conditions, the survival rate ranged between 13–15% in the range of 0.5–10 × 10^9 ^cells/ml.

To study the effect of cell density on transformation efficiency, cells of *L. lactis *LM0230 were collected, treated with LiAc and DDT, washed and diluted to various concentrations (Table 2). pTRKH3 was transformed, as described in the Methods section, into each cell concentration, and the transformed colonies were counted. Transformation efficiency varied directly with cell density (*P *< 0.001) (Table 2). The increase in transformation efficiency with higher cell densities appeared to be linear, suggesting that transformation was more efficient at increased cell densities and that high cell densities were critical to obtaining high transformation efficiencies in *L. lactis*. Previously, McIntyre and Harlander [5,6] made a similar observation for cell concentration and *L. lactis *transformation efficiency. However, the highest yield of transformants/μg DNA reported was in the order of 2.000 transformants/μg DNA corresponding to cell densities of 10^10 ^cfu/ml, which is extremely low compared to present results. A linear relationship between cell density and transformation efficiency has also been reported by Wu and Letchworth [16] with the yeast *P. pastoris*. An immediate explanation for this phenomenon is difficult since fewer DNA molecules were avalaible for each cell at high density. The possibility that cells electroporated at low densities failed to survive the procedure seems impossible since no difference was found in survival rates as mentioned above.

Following treatment with LiAc and DDT and electrotransformation, vector stability was examined in transformants of both tested strains containing pTRKH3. Transformants were cultivated for 25 generations in medium without erythromycin and subsequently plated on solidified medium with and without erythromycin. The vector was stably maintained since a loss of ≤ 4% after 25 generations without selection was observed. Structural stability of the plasmid was also examined by cultivation in selective medium for 25 generations, plating on solid medium and analysis of plasmid DNA from a number of colonies. Sequencing revealed no deletions indicating that pTRKH3 was also structurally stably maintained.

## Conclusion

Treatment of *L. lactis *spp. *lactis *LM0230 and *L. lactis *spp. *lactis *ATCC 11454 cells with LiAc and DDT prior to electrotransformation with plasmid pTRKH3, resulted in a tremendous improvement in transformation efficiency without affecting their survival rate. The developed methodology applied to high cell density suspensions (10^10 ^cells/ml) allows the production of large numbers of transformants. The described host-vector systems are reproducible and highly efficient.

## Methods

### Bacterial strains and growth conditions

*L. lactis *spp.*lactis *LM0230, a plasmid-free derivative of *L. lactis *C2 [5], and *L. lactis *spp.*lactis *ATCC 11454, a known nisin producer [17], were used in this study. *L. lactis *was grown on GM17 agar (Sharlau, Spain, in which lactose was replaced with 1% w/v glucose) at 30°C under 5% CO_2_. *Escherichia coli *K12JM110 harbouring the shuttle vector pTRKH3 (7766 bp; conferring tetracycline and erythromycin resistance) was propagated at 37°C on LB medium (Sharlau, Spain) containing 8 μg/ml tetracycline (Sigma) and 50 μg/ml erythromycin (Sigma).

### Plasmid vector pTRKH3

**Figure 1:**
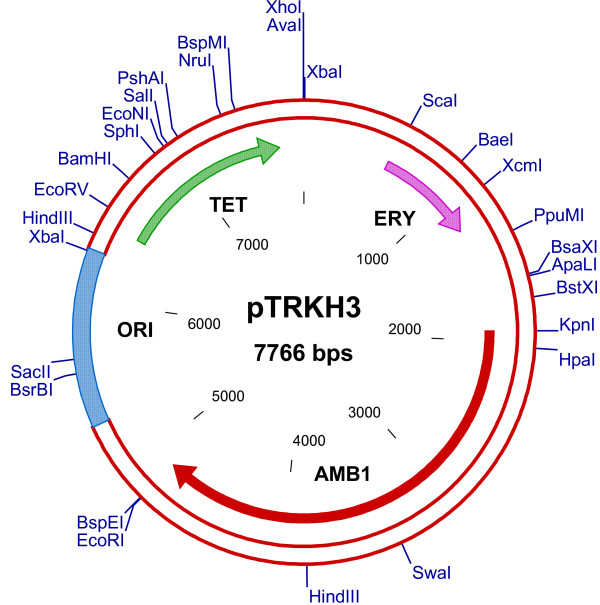
The shuttle vector pTRKH3.

The plasmid pTRKH3 was purchased from the BCCM/LMBP collection, University of Ghent, Belgium. pTRKH3 is a shuttle cloning vector for *E. coli*, *Lactococcus*, *Enterococcus*, *Streptococcus *and *Lactobacillus *species [17]. The vector has a medium copy number (30–40) in *E. coli*, and a high copy number (45–85) in streptococcal and lactococcal hosts. Tetracycline resistance is only expressed in *E. coli*, while erythromycin resistance is expressed both in *E. coli *and in Gram-positive bacteria. The restriction map of pTRKH3 is shown in Fig. 1.

### Plasmid DNA preparation

Plasmid DNA was extracted using the alkaline lysis procedure [21]. Extracted DNA was purified by cesium chloride-ethidium bromide density centrifugation [21]. DNA concentration was determined spectrophotometrically at 260 nm. Plasmid separation was done by agarose gel electrophoresis (SCIE-PLAS, UK), using Agarose Multi Purpose (MP) gels from Roche Applied Science (Germany) and 50 × TAE buffer (0.04 mol/l Tris-acetate, 0.002 mol/l EDTA, pH 8.0). Agarose gels were dried with a vacuum dryer (SCIE-PLAS Gel drier, GD4534) at 60°C for 40 min. Gels were viewed with tabletop UV emitter and photography of stained gels was carried out with a Vilber Lourmat (France) photodocumentation system (DD-001-FDC). DNA recovery from agarose was done using proprietary kits from Qiagen (Qiagen, CA, USA).

### Sequence analysis

The nucleotide sequence of pTRKH3, linearized by *Ava*I was determined by the primer walking strategy, starting from both ends. To cover the complete 7.766 bp, 35 primers were designed and each nucleotide was read at least two times in each direction. Sequencing was accomplished by using an Applied Biosystems model 373A automatic sequencer according to procedures provided by the supplier and fluorescent-dye-labeled dideoxyribonucleotides.

### Electroporation of Lactococcus lactis

Electroporation was done according to a modification of the method of Van der Lelie [9], proposed by Dornan and Collins [10]. Overnight cultures of *L. lactis *of both strains grown at 30°C under 5% CO_2 _in GM17 broth supplemented with 40 mmol/l threonine (Sigma) were diluted 1:12.5 in 25 ml of GM17. Cells were harvested by centrifugation at 10,000 g for 10 min when the optical density at 660 nm was between 0.26 and 0.38, according to Dornan and Collins [10]. Cell number was calculated according to McIntyre and Harlander [5,6]. The cells were washed sequentially with the following ice-cold solutions by alternate centrifugation and resuspension: bidistilled water 2.0 ml, bidistilled water 1 ml, 50 mmol/l EDTA 1 ml, bidistilled water 1 ml, 0.3 mol/l sucrose 1 ml, 0.3 mol/l sucrose 0.3 ml. After the final suspension, the cells were immediately electroporated at a concentration of 10^10 ^cells/ml, using an electroporator with pulse controller (Electro Cell Manipulator™ 600, BTX, USA). Electroporation was performed by a single pulse at 2.5 kV (*E *= 12.4 kV/cm), 200 Ω, and 25 μF (corresponding to pulse length of 4.6 ms), in 2 mm disposable electroporation cuvettes (CE-0002, Eurogentec, Belgium), using 1 μg of purified plasmid DNA. The cell suspension was diluted immediately by the addition of 5 ml GSM17 (GM17 containing 1% w/v sucrose) broth supplemented with 10 μg/ml erythromycin [5,6] and incubated for 2 h at 30°C under 5% CO_2 _before being placed on GSM17 agar supplemented with erythromycin (10 μg/ml). Appropriate controls confirmed the absence of transformants if either the electric pulse or the plasmid DNA was omitted. Transformation was confirmed by selection in erythromycin containing medium and plasmid analysis.

Statistical analysis were done by Student's *t*-test and regression analysis.

### Pretreatment of cells with LiAc and DTT

Pretreatment of *L. lactis *with lithium acetate and/or dithiothreitol was as follows: 10^9 ^cells were suspended at room temperature for 30 min in 8 ml of 100 mM LiAc, 10 mM DTT, 0.6 M sucrose, and 10 mM Tris-HCl, pH 7.5. Following treatment, the cells were pelleted, resuspended in 1.5 ml microcentrifuge tube, and washed as described above.

## Authors' contributions

Maria Papagianni conceived of the study, coordinated it, participated in its experimental part (microbial cultures, transformants studies) and drafted it. Nicholaos Avramidis worked on the electroporation protocols, sequencing and performed statistical analysis. George Filiousis worked on the electroporation protocols and carried out the electrophoresis. All authors read and approved the final manuscript.

## Figures and Tables

**Table 1:** DNA transformation efficiency of *L. lactis *spp. *lactis *cells with and without pretreatment with LiAc and DTT. Results are the mean (± SD) of 6 replications

LiAc (mM)	DTT (mM)	Transformants/μg DNA	
		*L. lactis *LM0230	*L. lactis *ATCC 11454
0	0	1.20 ± 0.50 × 10^5^	1.00 ± 0.48 × 10^4^
100	0	1.10 ± 0.52 × 10^5^	1.20 ± 0.50 × 10^4^
0	10	13.00 ± 8.02 × 10^6^	7.00 ± 5.01 × 10^5^
100	10	225.00 ± 52.51 × 10^7^	117.00 ± 42.55 × 10^6^

**Table 2:** Effect of cell density on transformation efficiency of *L. lactis *spp. *lactis *LM0230. Results are the mean (± SD) of 6 replications

Cells × 10^9^/ml	Transformants/μg DNA
0.5	5.00 ± 0.75 × 10^7^
1.0	22.00 ± 13.00 × 10^7^
2.5	53.60 ± 27.12 × 10^7^
5.0	162.00 ± 75.40 × 10^7^
10	221.00± 50.00 × 10^7^
